# Supplementary material for: Assessing Gut Microbiota in an Infant with Congenital Propionic Acidemia before and after Probiotic Supplementation
Source: Microorganisms. 2021 Dec 16;9(12):2599. doi: 10.3390/microorganisms9122599 (PMC8703847; doi:10.3390/microorganisms9122599)
Supplement: Supplementary file 1 [file microorganisms-09-02599-s001.zip › microorganisms-1463282-supplementary.pdf]

Assessing gut microbiota in an infant with congenital propionic acidemia before and after probiotic supplementation

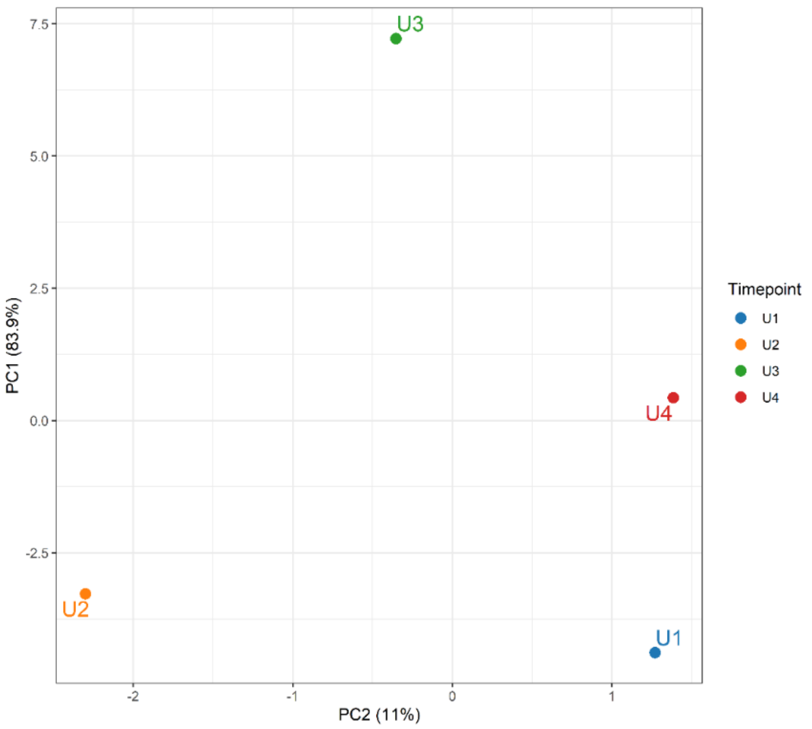

Supplementary Figure S1. Scatter plot of the first two principal components (PC1 and PC2) from the principal component analysis (PCA) of urine samples U1-U4.

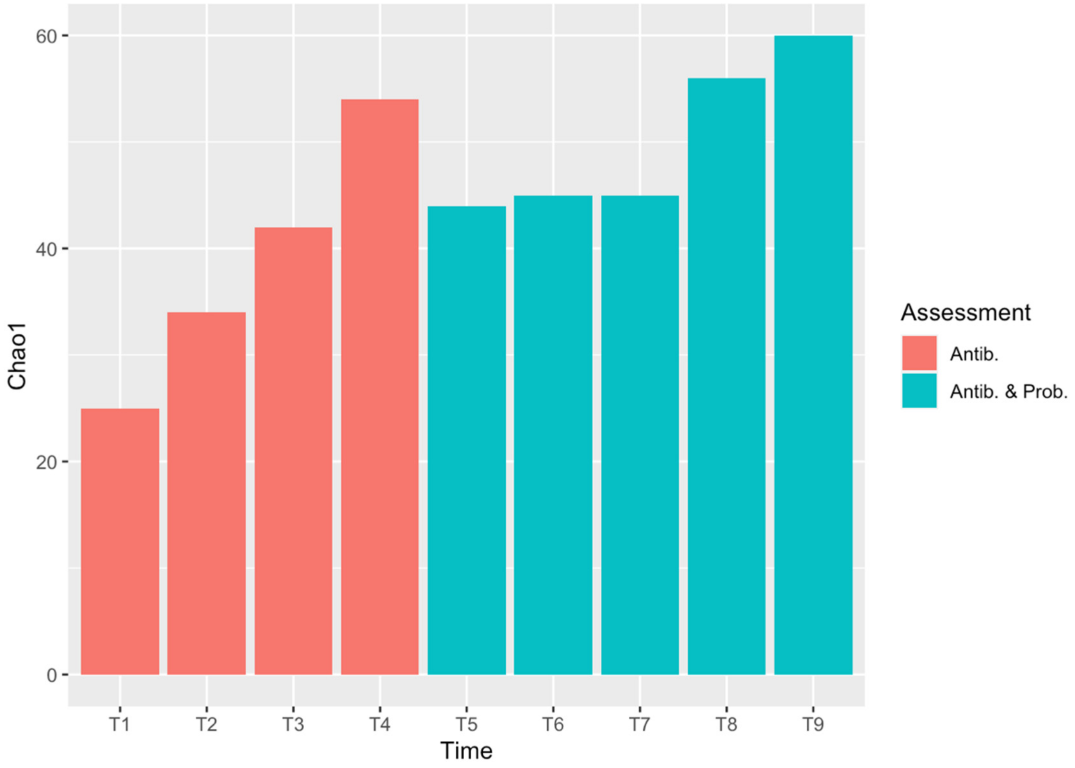

Supplementary Figure S2. Alpha diversity analysis of metabarcoding sequencing data. Alpha-diversity was performed using the "estimate_richness" function implemented in phyloseq estimated using the Chao1 metric. Salmon orange bars are related to samples T1-T4, collected at first month of life and associated to antibiotic treatment. Turquoise bars are related to samples T5-T9, collected at 4 months of life and associated to both antibiotic treatment and probiotic supplementation.

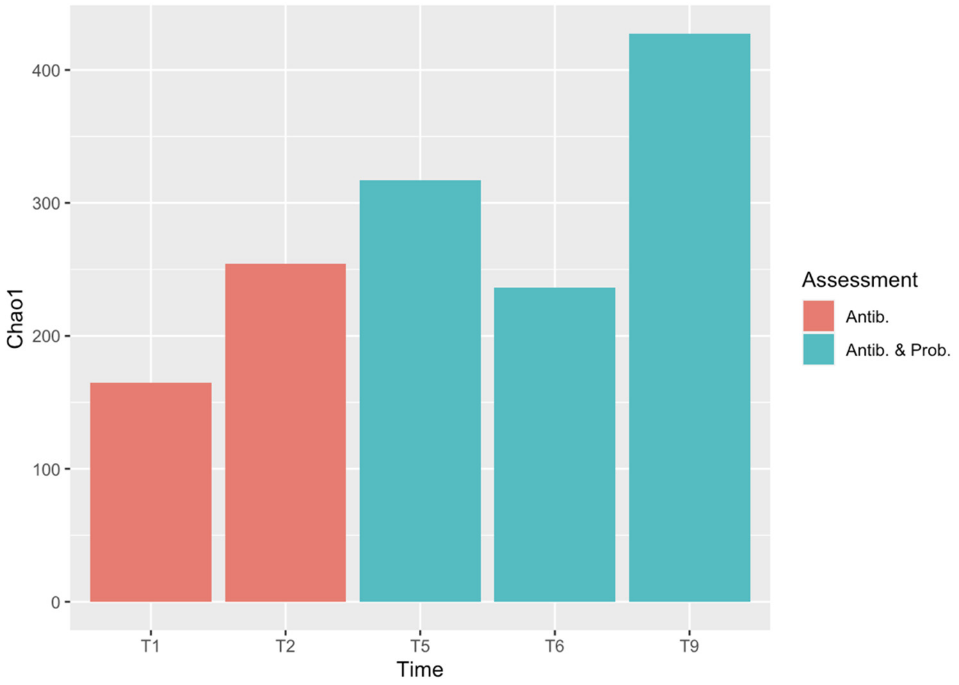

(a)

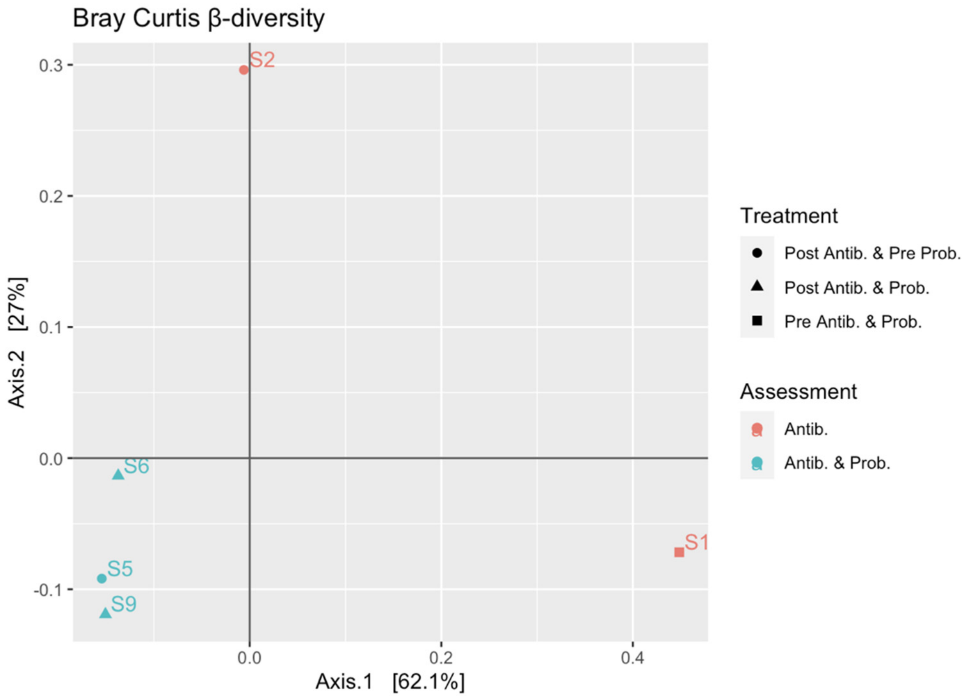

(b)

Supplementary Figure S3. Alpha (a) and Beta (b) diversity analysis of low coverage whole metagenome sequencing data. Alpha-diversity (a) was performed using the "estimate_richness" function implemented in phyloseq estimated using the Chao1 metric. Salmon orange bars are related to samples T1-T2, collected at first month of life and associated to antibiotic treatment. Turquoise bars are related to samples T5-T9, collected at 4 months of life and associated to both antibiotic treatment and probiotic supplementation. Beta diversity (b) rarefaction was based on Bray-Curtis distance metric using "plot_ordination". Shapes indicates the type of treatment: circles are related to samples collected after antibiotic treatment and before the probiotic supplementation (S2 and S5); triangles are related to samples collected after antibiotic treatment and probiotic supplementation (S6 and S9); squares are related to samples collected before antibiotic treatment and probiotic supplementation (S1). Colors indicate the two phases of the study: salmon orange is related to samples S1 and S2 collected at first month of life and associated to antibiotic treatment; turquoise is related to samples S5, S6 and S9 collected at 4 months of life and associated to antibiotic treatment and probiotic supplementation.

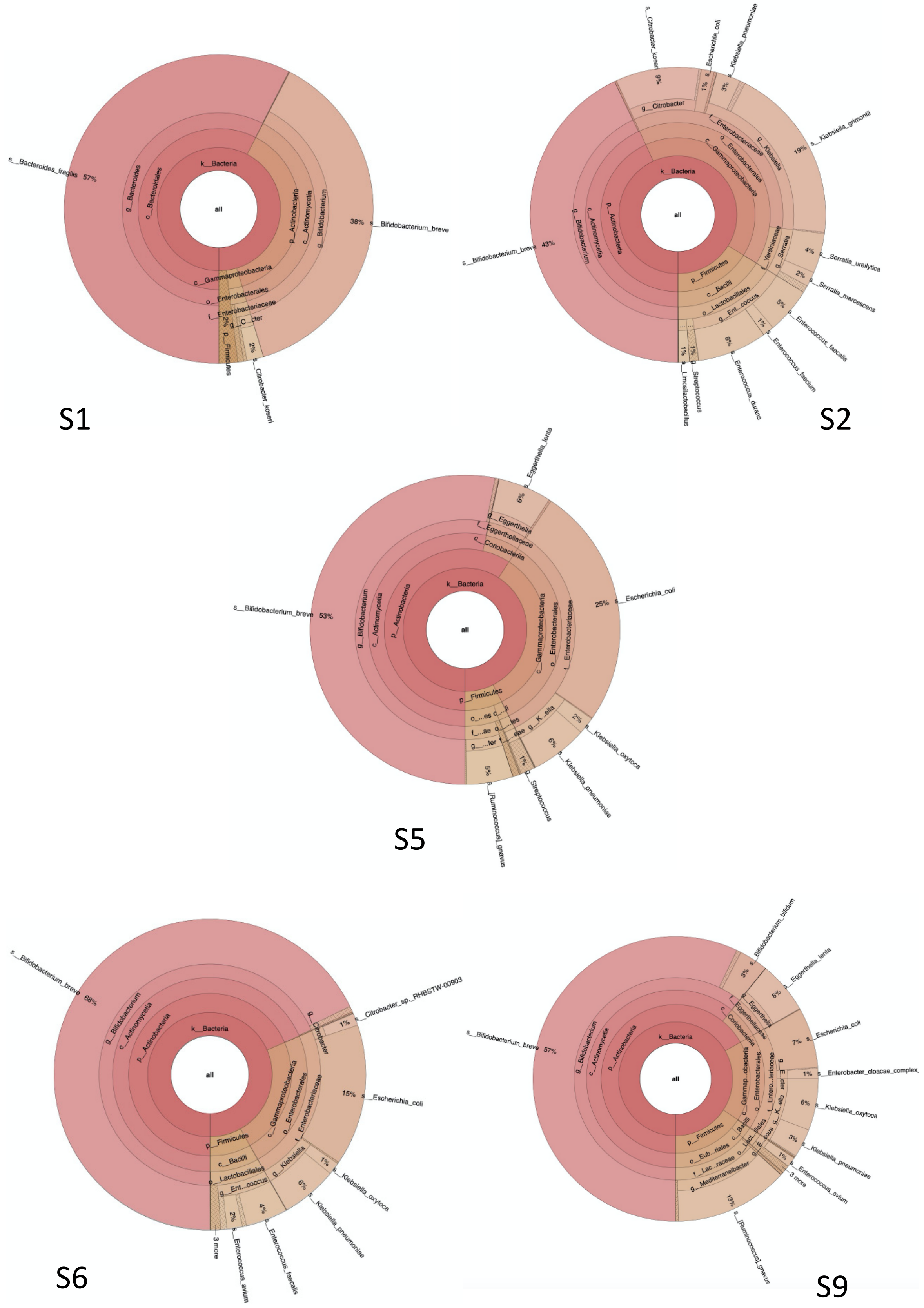

Supplementary Figure S4. Microbiota structure assessed with low coverage whole metagenome sequencing. Sample S1 was collected at 1st month of life before the antibiotic therapy, sample S2 was collected at 1st month of life 1 day after the end of the treatment; sample S5 was collected at 4 months of life before the 5th antibiotic therapy, sample S6 was collected at 4 months of life 1 day after the end of 5th antibiotic therapy and at first day of probiotic supplementation, S9 was collected at 4 months of life 21 days after the end of 5th antibiotic therapy and at the end of probiotic supplementation.

As shown also for metabarcoding data (see main manuscripit), *Bac. fragilis* dominated the gut microbiome in S1 with a relative abundance of 57%, followed by *Bif. breve* population (38%). Smaller fractions included members of *Proteobacteria* (*Citrobacter koseri*, 2%) and *Firmicutes* (2% of all), which were mostly represented by *Veillonella parvula* (1% of all) and members of *Lactobacillales* (mainly *Streptococcus* and *Enterococcus* spp.) (0,9% of all). In S2, *Bif. breve* was the most abundant member (43%), while *Bac. fragilis* was not detected. The *Enterobacteriaceae* represented the 34% of the total microbial population and it included members of genera *Klebsiella* (23%) (*K. oxytoca*, *K. michiganensis*, *K. pneumoniae*, *K. grimontii*, *K. variicola*), *Citrobacter* (*C. koseri*) (9%) and *Escherichia* (*E. coli*) (1%). Other *Proteobacteria* included *Serratia* species (*S. ureilytica*, *S. marcescens*) which represented 7% of the total population. Members of *Lactobacillales* constituted the 16% of the gut microbiota and they mainly comprised *Enterococcus* species (*E. durans*, *E. faecalis*, and *E. faecium*, with 8, 5 and 1% of total relative abundance, respectively), *Limosilactobacillus reuteri* (1%) and *Streptococcus* species (1%). As for S5, 59% of sample was covered by members of phylum *Actinobacteria*, and 53% was related to *Bif. breve* (0,6% of *Bif. longum* and 0,1% of *Bif. bifidum* were also detected) while the remaining 6% was related to *Eggerthella lenta*. *Enterobacteriaceae* spp. had a relative abundance of 33% and they were most represented by *E. coli* (25%) and *Klebsiella* spp. (*K. pneumoniae*, 6%; *K. oxytoca*, 2%); as for *Firmicutes* fraction (7% of all), it mainly included *Ruminococcus gnavus* (5% of all) and *Streptococcus* spp. (1%).

The relative abundance of *Actinobacteria* reached 69% in S6, with *Bif. breve* the most represented (68% of all). The population of *Enterobacteriaceae* slightly decreased compared to S5 (23%) while the *Firmicutes* (8% of all) mainly consisted of *Enterococcus faecalis* (4%), *Enterococcus avium* (2%) and *Streptcoccus* spp. (0,9%). Similarly to S6, sample S9 was characterized by 67% of *Actinobacteria* (57% of *Bif. breve*, 6% *E. lenta*, and 3% *Bif. bifidum*). The relative abundance of *Firmicutes* increased to 16% of all, with *Ruminococcus gnavus* the main representative (13%) followed by *E. avium* (1%) and *Streptococcus* spp. (0,8%). *Enterobacteriaceae* decreased to 17% and they mainly included *E. coli* (7% of all), *Klebsiella* spp. (9% of all) (*K. pneumoniae* and *K. oxytoca*) and *Enterobacter cloacae* (1%).
